# Impact of systematic MLC positional uncertainties on the quality of single‐isocenter multi‐target VMAT‐SRS treatment plans

**DOI:** 10.1002/acm2.13708

**Published:** 2022-06-22

**Authors:** Georgia Prentou, Eleftherios P Pappas, Eleni Prentou, Nikolaos Yakoumakis, Chryssa Paraskevopoulou, Efi Koutsouveli, Evaggelos Pantelis, Panagiotis Papagiannis, Pantelis Karaiskos

**Affiliations:** ^1^ Medical Physics Laboratory, Medical School National and Kapodistrian University of Athens Athens Greece; ^2^ Medical Physics Department Hygeia Hospital Athens Greece

**Keywords:** brain metastases, leaf offset, leaf positional uncertainty, MLC, single isocenter, spatial accuracy, SRS, stereotactic radiosurgery, VMAT

## Abstract

**Purpose:**

To study the impact of systematic MLC leaf positional uncertainties (stemming from mechanical inaccuracies or sub‐optimal MLC modeling) on the quality of intracranial single‐isocenter multi‐target VMAT‐SRS treatment plans. An estimation of appropriate tolerance levels is attempted.

**Methods:**

Five patients, with three to four metastases and at least one target lying in close proximity to organs‐at‐risk (OARs) were included in this study. A single‐isocenter multi‐arc VMAT plan per patient was prepared, which served as the reference for dosimetric impact evaluation. A range of leaf offsets was introduced (±0.03 mm up to ±0.30 mm defined at the MLC plane) to both leaf banks, by varying the leaf offset MLC modeling parameter in Monaco for all the prepared plans, in order to simulate projected leaf offsets of ±0.09 mm up to ±0.94 mm at the isocenter plane, respectively. For all offsets simulated and cases studied, dose distributions were re‐calculated and compared with the corresponding reference ones. An experimental dosimetric procedure using the SRS mapCHECK diode array was also performed to support the simulation study results and investigate its suitability to detect small systematic leaf positional errors.

**Results:**

Projected leaf offsets of ±0.09 mm were well‐tolerated with respect to both target dosimetry and OAR‐sparing. A linear relationship was found between *D*_95%_ percentage change and projected leaf offset (slope: 12%/mm). Impact of projected offset on target dosimetry was strongly associated with target volume. In two cases, plans that could be considered potentially clinically unacceptable (i.e., clinical dose constraint violation) were obtained even for projected offsets as small as 0.19 mm. The performed experimental dosimetry check can detect potential small systematic leaf errors.

**Conclusions:**

Plan quality indices and dose–volume metrics are very sensitive to systematic sub‐millimeter leaf positional inaccuracies, projected at the isocenter plane. Acceptable and tolerance levels in systematic MLC uncertainties need to be tailored to VMAT‐SRS spatial and dosimetric accuracy requirements.

## 1 INTRODUCTION

Linear accelerators (linacs) have shown notable technological advances and can be considered as high‐precision treatment delivery units that can achieve sub millimeter accuracy.^1^ Linac‐based stereotactic radiosurgery (SRS) is increasingly used nowadays in the treatment of multiple brain metastases cases.^2^, ^3^, ^4^ Considering that SRS involves the delivery of a high therapeutic dose in a single fraction and involves steep dose gradients, increased spatial accuracy is required. Total geometric inaccuracies of just a few millimeters could compromise target dose coverage, especially for cases with tiny brain lesions.^5^, ^6^, ^7^

Volumetric modulated arc therapy (VMAT) is implemented in clinical practice, as a contemporary SRS technique since it delivers precisely sculpted 3D dose distributions with up to 360‐degree rotation of the gantry in single‐ or multi‐ arc sessions. Single‐isocenter VMAT‐SRS treatment techniques were introduced for the concurrent treatment of multiple intracranial targets, offering further reduced treatment duration, while preserving high plan quality.^8^, ^9^, ^10^

However, single‐isocenter multi‐target VMAT‐SRS demonstrates increased sensitivity to geometric uncertainties^8^, ^9^, ^11^, ^12^, ^13^, ^14^, ^15^ compared to other approaches, and therefore, its efficacy partly relies on the overall spatial accuracy. VMAT delivery involves modulation of several mechanical parameters, such as dynamic multi‐leaf collimator (MLC), variable dose‐rate and variable gantry rotation speed.^16^, ^17^ The MLC mainly contributes to the beam shaping through the production of multiple segments per arc in order to deliver a uniform dose distribution to the targets, while sparing adjacent healthy tissue and organs‐at‐risk (OARs). Thus, leaf positioning accuracy is an important factor for safe and effective treatments and a stringent quality assurance (QA) program is required for the modeling and frequent verification of the MLC system.^1^, ^18^

Beam modeling in the Treatment Planning System (TPS) requires the determination of the most appropriate set of MLC parameters. Specifically for the Agility MLC system, combined with the Monaco TPS (ELEKTA, Crawley, UK), a vendor‐supplied QA package is provided to the physics staff in order to determine—among others—the “leaf offset” parameter. This is defined, as the deviation that may occur between the nominal (prescribed) leaf position and the actual value used for dose calculations by the TPS. The parameter is directly defined in millimeters (from −0.50 to +0.50 mm) and represents the absolute spatial deviation at the MLC plane.^19^ The leaf offset should be adjusted and used for dose calculations so that the prescribed in the TPS leaf positions match the corresponding ones obtained in the linac. An unnecessarily larger leaf offset results in an increased field size or segment width used for dose calculations. Thus, the leaf offset parameter is associated with the overall MLC positional accuracy and directly affects the output as well as the dose distribution for multiple small segments associated with dynamic VMAT plans. During commissioning of the linac's head, the standard, manufacturer‐recommended procedure for the determination of the most appropriate value relies on specially‐shaped test beams and film or detector array dosimetry.^19^ Analysis of the measured 2D dose distributions results in the most appropriate value for all MLC‐related modeling parameters in the TPS. The American Association of Physicists in Medicine (AAPM) TG‐106 report^20^ suggests the use of film, portal images, or diodes as measurement devices to determine the necessary MLC modeling parameters. Periodic checking and re‐adjustment are also needed to ensure the integrity of the controlling system. According to AAPM TG‐142 report,^21^ simple tests such as the picket fence test (described by LoSasso^22^) can assess positional accuracy qualitatively (by the matching of sequential segments and leaf transmission, particularly interleaf). The TG‐142 report recommends the picket fence test to be performed weekly with a careful examination of the image acquired by static film or on‐line portal image. On a monthly basis, an expansion of the leaf position accuracy test is recommended to account for gantry rotation which may affect leaf motion due to gravitational effects imposed on the leaf carriage system. The proposed tolerance value of leaf position accuracy on monthly basis is 1 mm for an Intensity Modulated Radiotherapy (IMRT) field, at the four cardinal gantry angles. Moreover, tolerance value of ±1 mm of leaf position repeatability is considered for annual testing. However, the above tolerance levels cannot be directly adopted for single‐isocenter multi‐target VMAT‐SRS procedures in which small fields and high dose gradients are commonly employed.

With respect to clinical plans, several groups have studied MLC offsets and their consequent impact on plan quality for various radiotherapy cases.^18^, ^23^, ^24^, ^25^, ^26^, ^27^, ^28^, ^29^ According to the work of Mu et al.,^27^ systematic errors of 1 mm were related to *D*_95%_ average changes of 8% for complex IMRT plans of head and neck cases, following a conventionally fractionated scheme. In another study,^30^ it was found that for nine‐beam conventional IMRT prostate treatments, a 1‐mm shift in all leaves of the MLC resulted in a mean target dose variation of approximately 6%. In a recent analysis of the direct clinical consequences of MLC positional errors in conventional VMAT therapy of glioma and glioblastoma cases using a 3D dosimetry system by Nithiyanatham et al.,^18^ it was found that the average change of *D*_95%_ to PTV for ±1 mm shift was 5.15%. Apparently for conventional IMRT and VMAT, shifts of 1 mm have a major impact on target dosimetry of various cases. Thus, for SRS treatments for challenging cases, it can be expected to have an even stronger impact on plan quality.

In an effort to estimate appropriate MLC positional uncertainty tolerance levels tailored to single‐isocenter multi‐target VMAT‐SRS requirements, the present work investigates the dosimetric impact of systematic leaf positional inaccuracies (induced by mechanical MLC leaf errors or sub‐optimal MLC modeling) on target dose coverage, conformality, and OAR‐sparing. To this end, single‐isocenter VMAT‐SRS treatment plans for five patients with three to four brain metastases are used. MLC leaf offsets of ±0.03, ±0.06, ±0.09, ±0.12, ±0.15, and ±0.3 mm (at the MLC plane) are simulated by varying the relevant MLC model parameter in the Monaco TPS. If projected at the isocenter plane, these offsets correspond to ±0.09, ±0.19, ±0.28, ±0.38, ±0.47, and ±0.94 mm for each leaf bank, affecting the field size or beam segments accordingly. A re‐calculation of the plan dose is performed in Monaco and the dosimetric impact on both targets and OARs is quantified and assessed using clinical dose–volume and plan quality metrics. A determination of relevant tolerance levels is attempted. Moreover, the commercially available StereoPHAN phantom in combination with the SRS mapCHECK 2D diode array (both produced by Sun Nuclear Corp., Melbourne, FL) were used for plan verification of a challenging VMAT‐SRS case and results are compared with TPS‐calculated dose distributions obtained after changing the clinically used leaf offset parameter. The aim of this experimental procedure is to support the results of the simulation study, but can also be regarded as a feasibility study for detecting small systematic leaf positional inaccuracies or sub‐optimal MLC modeling in the TPS.

## 2 METHODS

### 2.1 Targets and OARs contouring

In our previous publication,^31^ the effect of rotational patient setup errors on the quality of challenging VMAT‐SRS multiple brain metastases cases was studied. The same patient cohort is used for the purposes of the present work. Briefly, five patients with either three or four metastases (range 0.46–4.42 cc) treated concurrently were considered. For each case, at least one OAR lied in close proximity to a target (minimum distance of approximately 5 mm) but no target was tangent to an OAR. Contouring was performed in Monaco TPS version 5.11, (ELEKTA, Crawley, UK). Further details can be found in our previous study.^31^

### 2.2 Reference treatment plans

**FIGURE 1:**
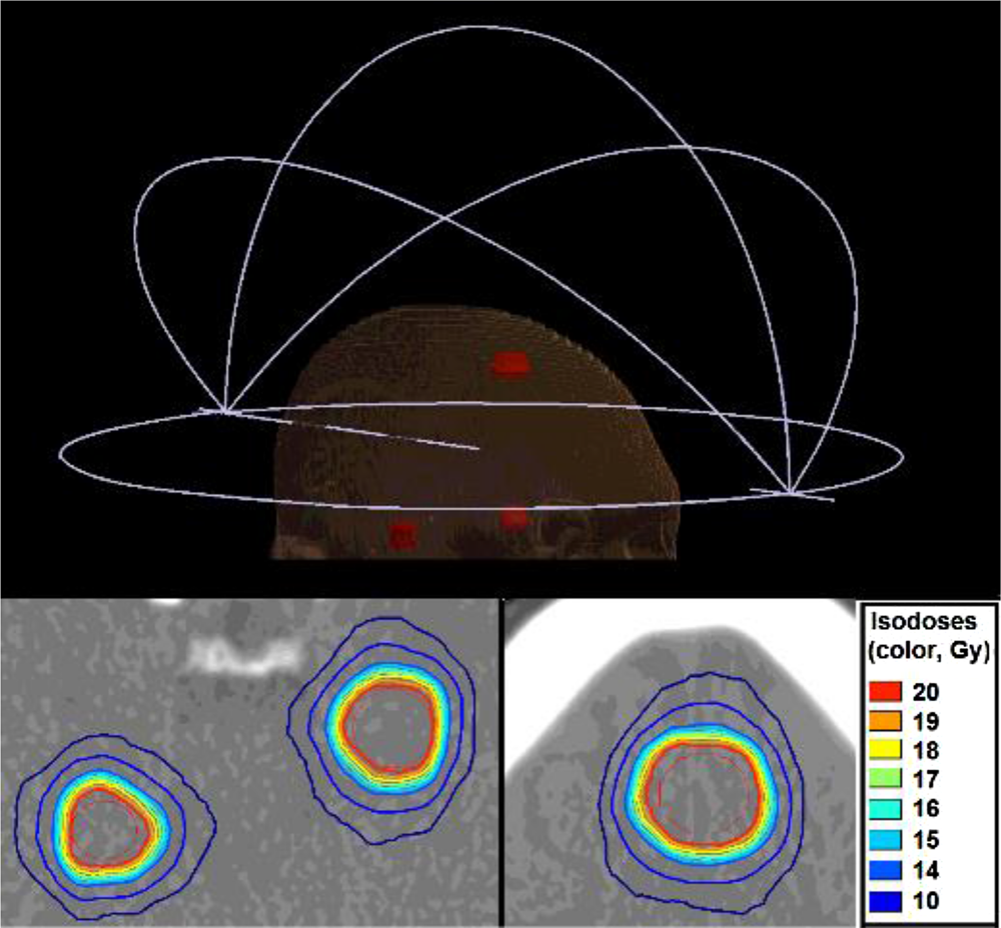
An indicative reference treatment plan (i.e., no leaf offset applied) involving three targets (metastases). Arcs configuration is presented (top) and isodose lines corresponding to reference dose distributions, are superimposed on axial slices (bottom) of the planning CT scan

Single‐isocenter multi‐target VMAT treatment planning was only considered. The isocenter was placed at the geometric center of all (three or four) targets. Non‐coplanar arcs were used with the following arrangement: a 360° arc (couch angle: 0°) and three half arcs (couch angles: 45°, 90°, 315°). Arcs configuration is graphically illustrated in Figure 1. An Agility linac (ELEKTA, Crawley, UK) with an MLC leaf width of 5 mm, and 6 MV flattening‐filter‐free (FFF) beams was used. For all cases, a dose of 20 Gy was prescribed in a single fraction. All dose calculations were performed using the X‐Ray Voxel Monte Carlo (XVMC) dose calculation algorithm^32^ with a uniform dose calculation grid resolution of 1 mm and statistical uncertainty of 1% per calculation.

Dose optimization was performed by prioritizing high target coverage, dose conformity, and steep dose gradients. Moreover, clinical dose constraints for all OARs involved were considered and strictly met in all cases. The constraints were given in detail in our previous publication (see Table 2 in Prentou et al.^31^). The aforementioned planning method has been repeatedly implemented in other independent studies.^33^, ^34^, ^35^

Resulting dose distributions were considered as the reference for investigating the impact of systematic MLC offsets on plan quality and dose–volume metrics.

### 2.3 MLC leaf offset simulation

In order to simulate and estimate the dosimetric effect of MLC leaf positional inaccuracies, a range of leaf offsets were introduced in the MLC geometry settings of Monaco TPS by varying the “leaf offset” parameter. Small and larger magnitudes of offsets were selected for simulation: ±0.03, ±0.06, ±0.09, ±0.12, ±0.15, and ±0.3 mm, defined at the MLC plane. A leaf offset of positive (negative) sign corresponds to a symmetrical opening (closing) of both leaf banks, leading to an enlargement (reduction) of the total field size and beam segments, if projected at the isocenter plane.

Taking into account that the focus‐to‐MLC distance in the Agility linac head^36^ is equal to 31.8 cm and that the source‐to‐isocenter distance for isocentric techniques is 100 cm, the projected systematic leaf positional offsets, at the isocenter plane, were calculated for all MLC leaf offsets introduced. More specifically, the projected leaf positional offsets were found approximately equal to: ±0.09, ±0.19, ±0.28, ±0.38, ±0.47, and ±0.94 mm for the respective leaf offsets at the MLC plane. Each offset is applied to both leaf banks simultaneously, affecting the beam segments. To better illustrate the magnitude of the offsets simulated, if static conventional fields were used, these offsets would correspond to a total change of the field size at the isocenter plane of twice the value of the projected leaf positional offset (i.e., ±0.19, ±0.38, ±0.57, ±0.75, ±0.94, and ±1.89 mm, respectively). Selection of the range of projected leaf offsets was based on the grounds that geometric uncertainties of the degree of 1 mm at the isocenter plane are expected to impact considerably the plan quality of VMAT‐SRS.^7^, ^31^, ^37^, ^38^

For each simulated leaf offset, the original (reference) plan was saved as a new plan in Monaco, with identical planning parameters, except for the dose distribution, which was re‐calculated for every applied offset, serving as the evaluated dose distribution. Twelve new plans per patient were created and evaluated, and 60 new plans in total.

### 2.4 Plan evaluation tools

Clinically used dose–volume metrics for targets and OARs were calculated for both reference and evaluated dose distributions, (e.g., the *D*_max_ [the maximum dose delivered to a structure] and the *V*_xGy_ [the volume of a structure receiving at least × Gy]). Dose–volume histogram (DVH) analysis was performed for all structures involved. Target dose conformity indices, such as Paddick' s conformity index (PCI)^39^ and Paddick' s gradient index (PGI),^40^ were also calculated. It is noted that PCI is affected by both target coverage and prescription isodose volume outside the target thus also affecting the surrounding critical organs dosage. The impact of projected leaf positional offsets was quantified by comparing reference and evaluated dose distributions in terms of the above plan quality and dose–volume metrics.

Moreover, all clinical dose constraints for OARs (see Section 2.2 and table 2 in Prentou et al.^31^), which were strictly met in the reference plans, were also calculated for the evaluated dose distributions. Potential violations were identified and corresponding plans (i.e., projected leaf offsets) were marked as potentially clinically unacceptable.

### 2.5 Experimental verification

**FIGURE 2:**
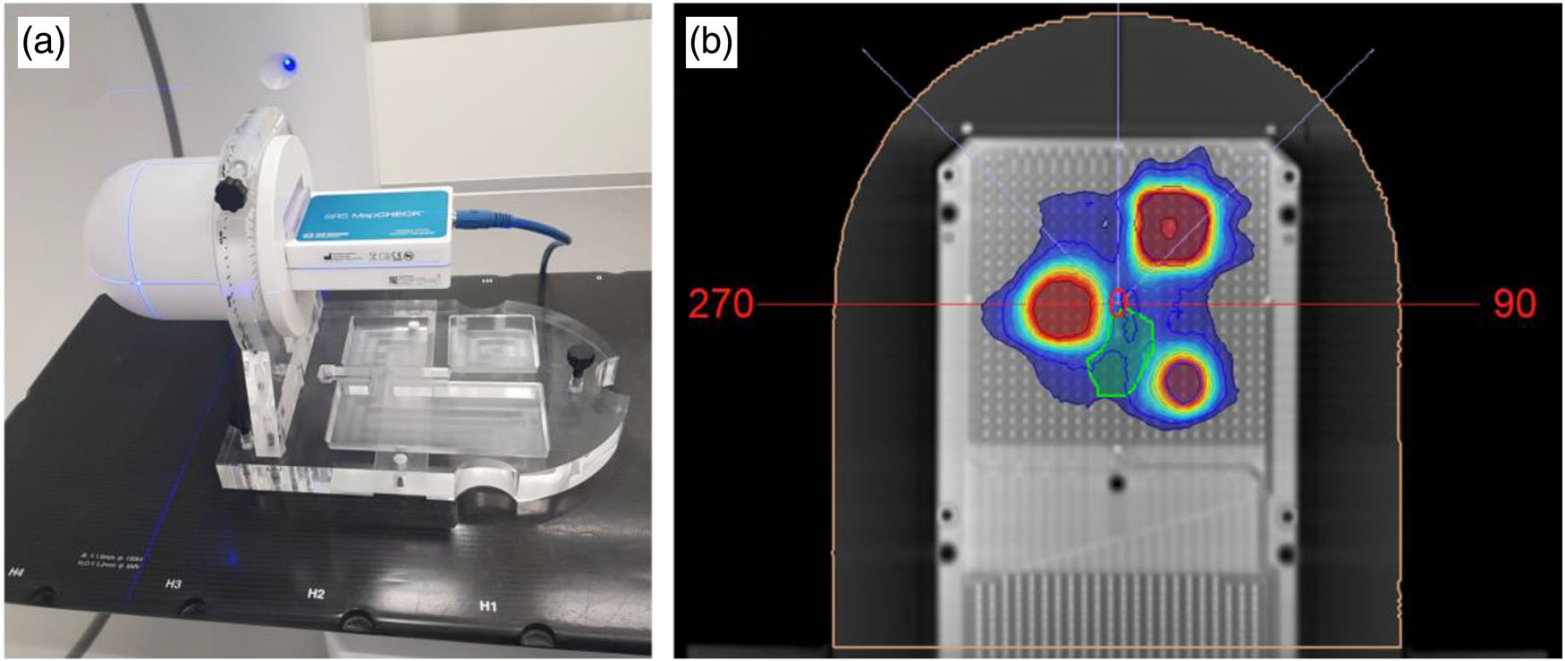
(a) The StereoPHAN phantom and the SRS mapCHECK diode array positioned on the couch for CT scanning. (b) An axial CT slice of the diode array with the TPS‐calculated dose distribution corresponding to the clinically used leaf offset parameter. Contours legend: targets: red, blue, and purple; brainstem‐like critical organ: green

Experimental dosimetry was also performed in order to support the findings of the simulation study, as well as demonstrate a simple QA check to detect systematic leaf positional inaccuracies or sub‐optimal MLC modeling. In specific, the StereoPHAN phantom in combination with the SRS mapCHECK diode array (both Sun Nuclear Corp., Melbourne, FL) was used in a plan QA procedure. The dosimetric system employed has been repeatedly described and evaluated for SRS plan QA.^41^, ^42^, ^43^ Briefly, the StereoPHAN is an acrylic‐based phantom, cylindrical in shape (diameter of 15.24 cm) with a hemispherical tip (Figure 2a). The total length of the phantom is 20.87 cm. The SRS mapCHECK is an array of 1013 n‐type diode detectors (0.007 mm^3^ each), spaced every 2.47 mm and centered on a 77 × 77 mm^2^ plane. The array is enclosed in a 320 × 105 × 45 mm^3^ acrylic slab (Figure 2a). The phantom‐detectors system can rotate around its central axis, enabling measurements on a user‐selected plane.^41^, ^42^, ^43^

The phantom was CT‐scanned at 120 kVp with the array of diodes aligned with the coronal plane, as shown in Figure 2a. Imaging parameters were identical with the ones used for patient scanning for SRS treatment planning. Images were imported to Monaco TPS, and three hypothetical targets with volumes similar to those used in the simulation study (0.53, 2.25, and 4.03 cm^3^) were contoured on the plane of detectors (Figure 2b). A brainstem‐like OAR was also contoured in the vicinity of the two targets in order for the case to resemble a clinical one used in the simulation study (Figure 2b). Plan optimization was performed considering clinical dose constraints and target dosimetry goals. Prescription dose, calculation algorithm, and grid resolution were described in Section 2.2. In addition to the clinically used leaf offset parameter, the dose distribution was re‐calculated after changing its value in Monaco, without re‐optimizing the plan. The applied changes to the clinically used leaf offset parameter were identical to the ones considered in the simulation study. All calculated dose distributions, contours, and dose delivery data were exported from the TPS in dicom file format.

Dose delivery involved four non‐coplanar arcs (as in the simulation study) and was carried out by a clinical VersaHD 6MV FFF linac (ELEKTA, Crawley, UK), equipped with an Agility treatment head. Prior to measurements, the SRS mapCHECK diode array was calibrated by implementing the absolute and relative calibration procedures, employing standard radiation fields, as recommended by the manufacturer and described in Ahmed et al.^41^

Measurements were recorded and analyzed by the SNC Patient QA software v.8.4.1.2 (Sun Nuclear Corp., Melbourne, FL). The software corrects detectors’ readings to account for pulse repetition rate, diode temperature, and angular dependence effects, using plan data processed from the dicom files.^42^, ^43^ The obtained 2D absolute dose distribution was compared against all TPS‐calculated ones, corresponding to the clinically used leaf offset value and the biased ones. The software also allows for comparisons using the global gamma Index tool (GI). GI passing rates were obtained for 3%/1 mm dose difference and distance‐to‐agreement passing criteria, respectively.^41^, ^42^, ^43^ A dose cut‐off threshold of 10% of the maximum dose was applied to exclude measurements of very low dose from the GI analysis.

## 3 RESULTS

### 3.1 Dosimetric impact on target dosimetric indices

**FIGURE 3:**
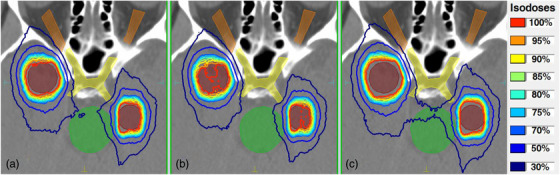
Axial CT slice with isodose lines (%) superimposed corresponding to (a) the reference plan, as well as plans with a systematic leaf positional offset of (b) −0.47 mm and (c) +0.47 mm, projected at the isocenter plane. Isolines are normalized to the prescription dose (i.e., 20 Gy). Contours legend: targets: maroon; brainstem: green; optic chiasm: yellow; optic nerves: brown

Figure 3a presents the reference dose distribution for two targets and surrounding OARs (brainstem, optic chiasm, and optic nerves) for an indicative case. Corresponding dose distributions after applying leaf positional offsets of −0.47 mm and +0.47 mm, projected at the isocenter (simulated by applying ±0.15 mm leaf offsets at the MLC plane), are shown in Figures 3b and 3c, respectively. In Figure 3b, the prescription isodose does not conform well around the targets shapes, resulting in poorer target coverage. Accordingly, in Figure 3c, PCI is compromised as the volume covered by the prescription isodose is considerably increased.

**FIGURE 4:**
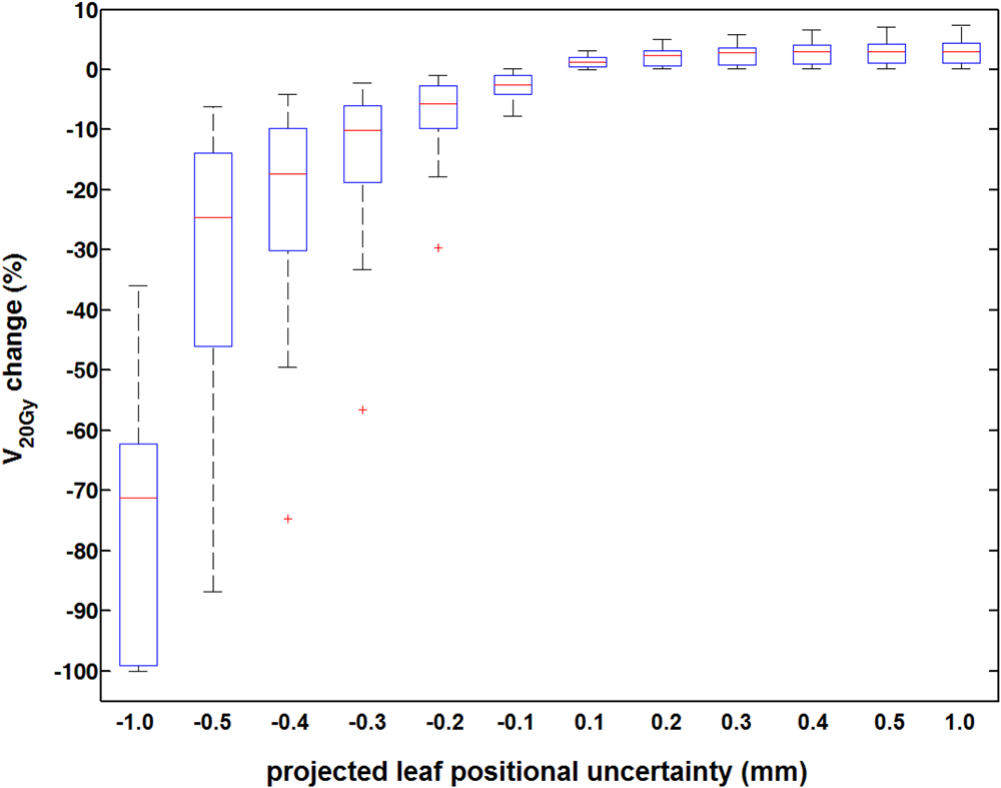
Box and whisker plots summarizing *V*_20Gy_ deviations induced by projected leaf positional offsets for both negative and positive signs. Red lines indicate the median of the data, while boxes range from the 1st to 3rd quartile. Whiskers depict the remaining data or extend up to 1.5 times the interquartile range in either direction. Red marks denote any outliers

For all cases and 18 targets considered, box and whisker plots related to percentage changes of target coverage (*V*_20Gy_) as a result of all projected leaf offsets applied, are shown in Figure 4. For offsets of negative sign, the dosimetric impact is more severe due to the induced field size and beam segment reduction. Minimal *V*_20Gy_ changes (<10%) are noticed for the smallest (±0.09 mm) projected leaf offsets. Considerable target coverage loss (>5%) occurs for −0.19 mm projected leaf offset for the majority of targets. All larger negative offsets resulted in median changes above 10% that increase as leaf offset increases (Figure 4).

**FIGURE 5:**
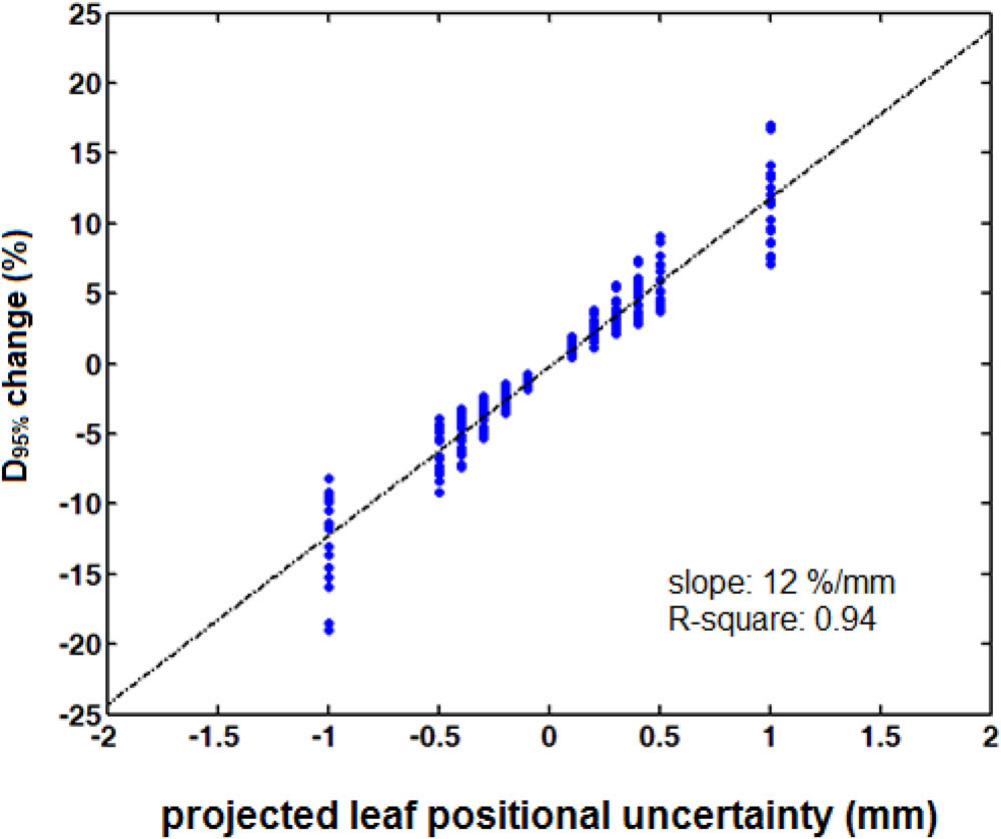
D_95%_ percentage change plotted against projected leaf positional offset for all patients and metastases (i.e., a total of 18 targets) considered. Fitted linear trendline along with calculated slope and R‐square is also shown

Target dosimetry susceptibility to projected leaf positional offsets is also quantified in Figure 5, where changes to *D*_95%_ (with respect to reference plans) are plotted against the projected offset applied. Considerable deterioration of *D*_95%_ (>5%) is observed for a 0.4 mm offset irrespective of the sign of positional uncertainty assumed. A fitted linear trendline is also given (Figure 5).

Reference and evaluated dose distributions are compared in terms of PCI and PGI in Table 1. Both indices were found susceptible to leaf offsets. Adopting a threshold of 10% median change (among all 18 targets) in PCI and PGI, projected offsets of +0.19 mm and +0.28 mm, respectively, are tolerated. In general, PCI is more sensitive to leaf offsets than PGI.

**TABLE 1:** The maximum and median deviations (with respect to reference plans) for PCI, and PGI for all 18 targets and five patients considered and all simulated leaf positional offsets of positive sign, projected at the isocenter plane

**Metric**	**Projected leaf positional offset (mm)**	**Median change (%)**	**Maximum change (%)**
PCI	0.09	−0.7%	−4.3%
	0.19	−3.7%	−9.0%
	0.28	−7.0%	−13.8%
	0.38	−12.0%	−17.8%
	0.47	−16.0%	−21.9%
	0.94	−31.0%	−37.9%
PGI	0.09	−0.1%	−4.0%
	0.19	−5.7%	−7.4%
	0.28	−8.5%	−9.9%
	0.38	−10.2%	−12.7%
	0.47	−11.5%	−15.0%
	0.94	−18.1%	−23.2%

Abbreviations: PCI, Paddick's conformity index; PGI, Paddick's gradient index.

**FIGURE 6:**
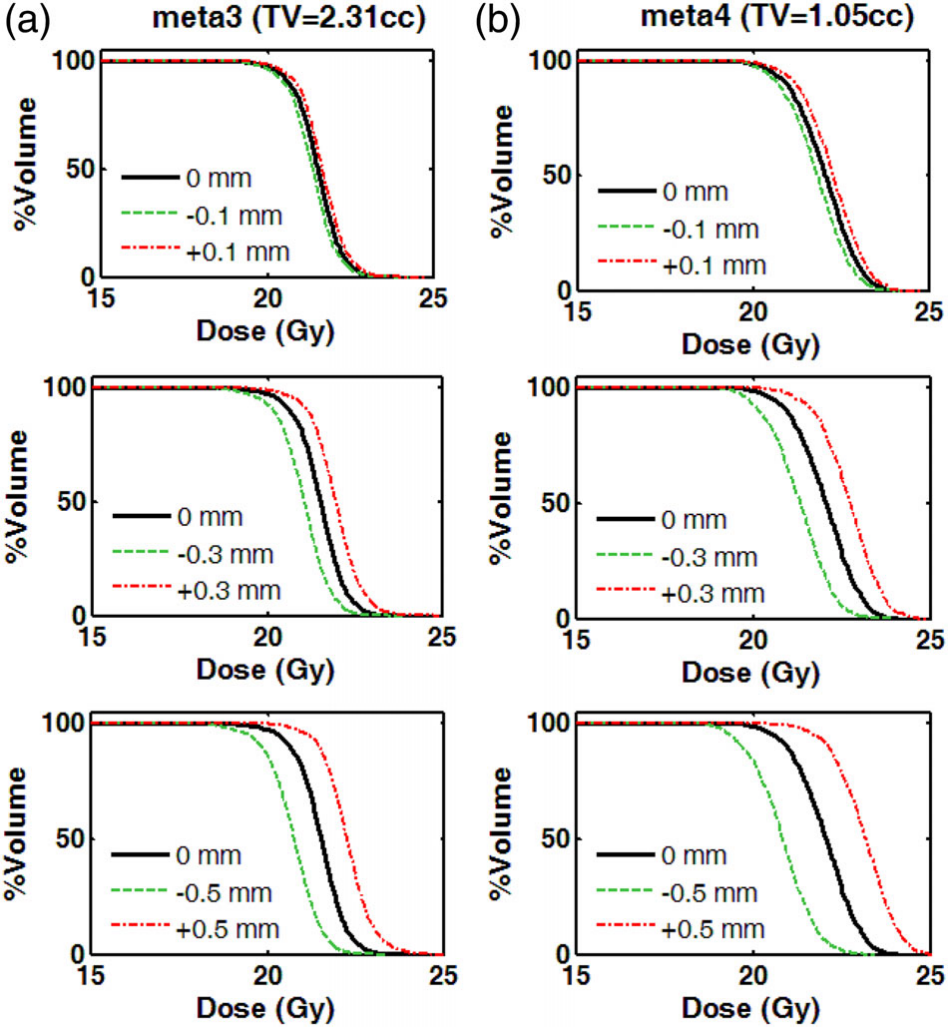
Calculated DVHs of a case involving a total of four targets. (a) A large target (2.31 cc) and (b) a smaller target (1.05 cc) are indicatively shown here. For each target, the DVH corresponding to the reference plan (i.e., no offset applied) is presented (solid black line) as well as the obtained DVHs following introduction projected leaf positional offsets of positive (red dash‐dotted line) and negative (green dashed line) signs. The magnitudes of leaf positional offsets are (from top to bottom) ±0.09, ±0.28, and ±0.47 mm, projected at the isocenter plane. TV: target volume; DVH: dose–volume histogram

To highlight the susceptibility of target dosimetry plan quality metrics with respect to target volume, Figure 6 presents DVHs for a fairly large (2.31 cc) and a smaller lesion (1.05 cc, same patient), calculated for the reference plan and projected leaf offsets of ±0.09, ±0.28, and ±0.47 mm (by applying offsets of ±0.03, ±0.09, and ±0.15 mm, at the MLC plane, respectively). For the larger target volume (Figure 6a), the induced effect is always larger in magnitude compared to dosimetric impact for the smaller target volume (Figure 6b), but still significant for projected offsets ≥0.28 mm. The effect of projected leaf positional offsets of 0.09 mm can be considered negligible, since changes of the DVH metrics are hardly noticed for the majority of targets.

### 3.2 Dosimetric impact on OAR‐sparing

Regarding OARs lying in the vicinity of targets, such as the brainstem, optic chiasm, and optic nerves, maximum doses are either increased or decreased depending on the magnitude, and direction of the leaf offset simulated, as well as the relative locations of the neighboring targets. As expected, projected leaf offsets of the same magnitude but different direction led to changes of the same order but different sign of the dose volume metrics for OARs.

Qualitatively, the dosimetric impact of leaf positional errors on OAR‐sparing is illustrated in Figure 3, for an indicative case and simulated projected offsets of ±0.47 mm. For the reference plan (Figure 3a), the 30% and 50% isolines are fairly tight around the two targets. If a systematic positive leaf offset is introduced (Figure 3c), the volume enclosed by all isodoses considerably expand. The consequent dosimetric burden is evident for the brain parenchyma, as well as the brainstem and the optic chiasm.

In a more quantitative analysis, Table 2 lists median and maximum percentage changes (among all patients and targets) of dose–volume metrics considered in clinical practice, induced by all the simulated leaf positional offsets of positive sign with respect to values of the reference plans. All OARs, including the brain parenchyma, were found extremely sensitive to projected leaf positional offsets. Indicatively, *D*_max_ delivered to the optic nerve can increase up to 8.4% even for projected errors of as small as +0.19 mm (Table 2). The smallest magnitude of simulated projected offset (0.09 mm) was related to minimal changes (<5%) of *D*_max_ and no violation of dose constraints was observed.

**TABLE 2:** The maximum and median deviations (with respect to the reference plans) for dose–volume metrics for OARs and all patients considered. Leaf positional offsets (projected at the isocenter plane) of positive sign are only included

**OAR**	**Metric**	**Projected leaf offset (mm)**	**Maximum change (%)**	**Median change (%)**
Brainstem	*D*_max_	0.09	2.1	0.6
		0.19	4.8	3.1
		0.28	7.4	4.8
		0.38	9.0	5.8
		0.47	12.4	7.3
		0.94	24.9	16.2
	*D*_0.02cc_	0.09	3.0	0.8
		0.19	6.1	5.5
		0.28	9.7	7.5
		0.38	11.6	9.6
		0.47	14.3	11.1
		0.94	28.5	21.9
Optic chiasm	*D*_max_	0.09	3.6	2.4
		0.19	7.5	5.5
		0.28	9.9	6.9
		0.38	11.6	10.7
		0.47	14.8	12.0
		0.94	26.8	20.1
	*D*_0.02cc_	0.09	5.8	2.2
		0.19	5.8	5.0
		0.28	7.8	5.3
		0.38	10	7.5
		0.47	12.7	10.6
		0.94	25.1	20.9
Optic nerve	*D*_max_	0.09	5.0	0.4
		0.19	8.4	3.0
		0.28	8.9	3.9
		0.38	11.2	5.2
		0.47	11.2	5.8
		0.94	31.4	12.6
	*D*_0.02cc_	0.09	3.3	1.8
		0.19	5.3	4.6
		0.28	6.1	5.3
		0.38	9.5	7.3
		0.47	12.0	7.1
		0.94	22.4	15.6
Brain parenchyma	*V*_7Gy_	0.09	3.1	3.0
		0.19	6.6	6.0
		0.28	10.5	9.0
		0.38	14.0	12.2
		0.47	17.8	15.3
		0.94	38.4	32.4
	*V*_12Gy_	0.09	4.1	3.7
		0.19	8.5	7.4
		0.28	12.8	10.8
		0.38	17.2	14.5
		0.47	21.6	18.1
		0.94	44.0	37.4

Abbreviations: *D*_0.02cc_, minimum dose delivered to 0.02 cc of the structure; *D*_max_, maximum dose; OARs, organs‐at‐risk; *V*_xGy_, volume of structure receiving at least x Gy.

According to the results presented in Table 2, compromised OAR‐sparing might be realized if the leaf offset parameter is not carefully accounted for or long‐term variations occur. The increased dose delivery to critical organs in several cases resulted in dose–volume indices exceeding the original dose constraints considered (and strictly met) during reference treatment planning, and plans that could be considered clinically unacceptable were obtained even for a projected leaf offset of as small as 0.19 mm. In specific, dose constraint violation occurred for the optic chiasm and optic nerve in two (out of five) cases for the 0.19 mm projected offset.

### 3.3 Experimental verification

**FIGURE 7:**
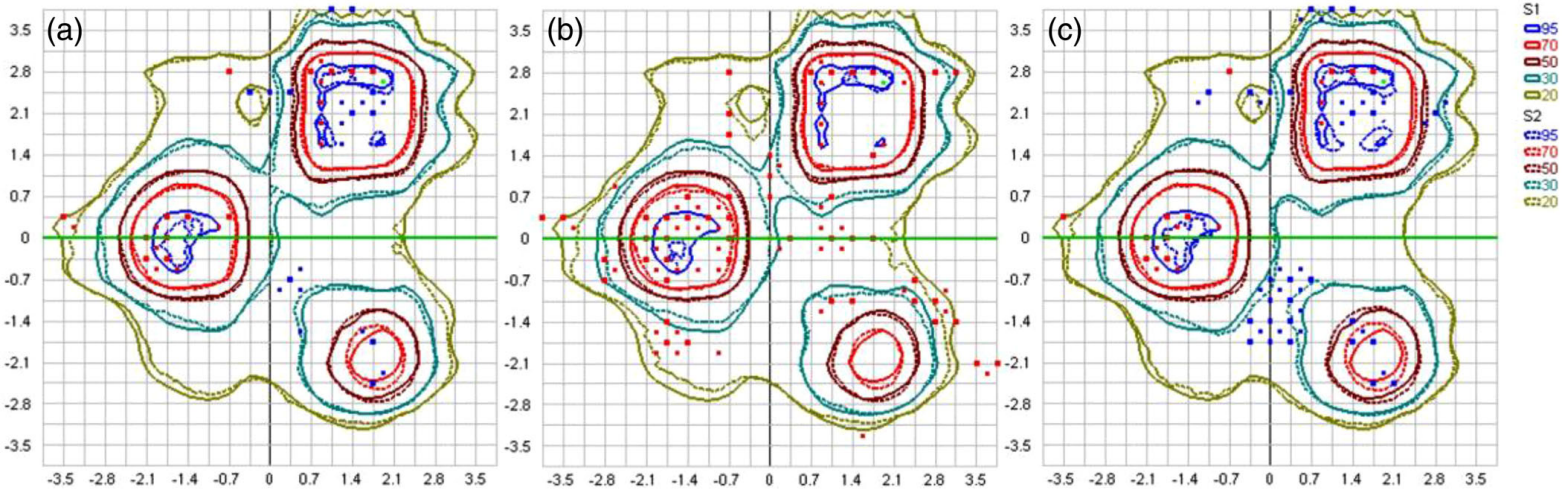
The measured (continuous isolines) dose distribution compared against TPS calculations (dashed isolines) on the plane incorporating the array of diodes for (a) the clinically used leaf offset value, and after applying a change of (b) −0.15 mm and (c) +0.15 mm, introducing a leaf positional offset of −0.47 mm and +0.47 mm, projected at the isocenter plane, respectively. Although absolute dose measurements were performed, distributions presented here are relative to the local maximum dose. Figures created using the SNC Patient QA software

In Figure 7a, measured 2D dose distributions are compared against TPS calculations obtained for the clinically used leaf offset value, for the entire measurement plane. A high‐level of agreement is achieved, at both low and high dose areas. GI passing rate using 3%/1 mm criteria and 10% cut‐off threshold reached 95.4% (Table 3). For the same treatment plan, Figure 7b,c presents isodose lines for the measured distribution and corresponding TPS‐calculated ones, after changing the leaf offset value in the TPS by −0.15 mm and +0.15 mm, which correspond to an introduction of leaf positional errors (compared to the clinically used setting) by −0.47 mm and +0.47 mm, projected at the isocenter plane. Accuracy of TPS dose predictions is evidently compromised in both positive and negative offset cases. This is also verified by the calculated GI passing rates (Table 3).

**TABLE 3:** Experimental verification study results. Global Gamma Index (GI) passing rates for 3%/1 mm passing criteria using a 10% threshold of the maximum dose. The 2D absolute dose measurements always serve as the reference distribution for the evaluation of TPS‐calculated ones (with or without changing the clinically used leaf offset parameter). Corresponding offsets projected at the isocenter plane are also given. GI values were determined by the SNC Patient QA software

**Leaf offset parameter change (mm)**	**Projected leaf positional offset change (mm)**	**GI passing rate (%) with criteria 3%/1 mm**
−0.30	−0.94	79.2
−0.15	−0.47	89.9
−0.12	−0.38	91.0
−0.09	−0.28	92.9
−0.06	−0.19	94.1
−0.03	−0.09	94.7
0	0	95.3
+0.03	+0.09	94.9
+0.06	+0.19	94.8
+0.09	+0.28	93.9
+0.12	+0.38	93.1
+0.15	+0.47	92.7
+0.30	+0.94	89.6

To better highlight the dosimetric system's sensitivity to detect small positional inaccuracies, GI passing rates are given in Table 3 for all changes made to the clinically used leaf offset value. The dose distribution obtained for the clinically used leaf offset exhibits the highest GI passing rate. Applying a change of >0.2 mm in magnitude (projected at the isocenter plane) evidently results in a considerable reduction of the calculated GI passing rate (Table 3).

## 4 DISCUSSION

Overall results of this work suggest that depending on the degree and direction of systematic MLC positional errors, compromised efficiency in VMAT‐SRS procedures might occur, especially if tiny lesions are involved and/or OARs lie in close proximity to targets.

Regarding target dosimetry, several groups^18^, ^24^, ^25^, ^27^, ^29^, ^30^ have evaluated the MLC accuracy and its dosimetric effect but conventionally fractionated radiotherapy was only considered (see Section 1 for more details). Specific to SRS procedures, Denton et al.^44^ quantified isocenter measurements to establish clinically meaningful thresholds based on the fundamental limitation of linac isocentricity. The MLC offset was investigated as an individual contributor to uncertainty in isocenter definition. It was found that variations in positioning of the test tool constituted, on average, 0.38 mm magnitude of correction, and MLC offset contributed by 0.16 mm. In that study, MLC offset was defined as the degree of misalignment of the MLCs with respect to the collimators. In another publication, Lee et al.^45^ investigated the effects of the static dosimetric leaf gap (DLG) parameter on MLC‐based small‐field dose distributions for intensity‐modulated radiosurgery. The results of their study showed that dose differences up to 30.8% were observed when the actual DLG deviated from the reference value by 1 mm. Thus, DLG is an MLC model parameter which strongly affects SRS treatment efficiency.

In the present study, the impact of MLC leaf offset parameter on target dosimetry was mainly based on *V*_20Gy_ and *D*_95%_ results (Figures 4 and 5, respectively). Systematic errors of ±0.09 mm projected at the isocenter plane (originating from ±0.03 mm leaf offsets at the MLC plane) are well tolerated. Moreover, it was found that the *D*_95%_ percentage dose change has a linear relationship with MLC leaf positional uncertainty (Figure 5) which was also verified in some of the aforementioned studies.^18^, ^24^, ^25^, ^29^ Increasing *D*_95%_ with increasing field size for the same plan and MUs implies that the contribution of scatter radiation to target dose increases considerably. However, results of the present work demonstrate increased sensitivity to systematic MLC leaf positional inaccuracies as compared to the literature. This can be attributed to the nature of VMAT‐SRS plans, studied herein, involving smaller fields and beam segments, steeper dose gradients and tiny lesions, as opposed to conventionally fractionated IMRT techniques.

According to the findings of Mu et al.,^27^ systematic MLC positional errors of 1 mm were related to average changes of dose–volume metrics of OARs, such as the brainstem, of 12%, for complex IMRT plans of head and neck cases, following a conventionally fractionated radiotherapy scheme. Rangel et al.^24^ also found that systematic MLC positional errors of 1 mm led to a brainstem average equivalent uniform dose (EUD) change of (3.22 ± 1.06) Gy with respect to the prescribed EUDs. It was also reported that if adopting a 2‐Gy change for the OARs as acceptable level of deviation in dose due to MLC effects only, systematic leaf positional uncertainties will need to be limited to 0.3 mm. According to the study of Nithiyanatham et al.,^18^ the average deviations of *D*_max_ for systematic MLC positional errors of ±1, ±0.5, and ±0.3 mm were 5.4%, 2.8%, and 0.83% for the brainstem.

Based on the results of the present study (Table 2 and Section 3.2), tolerances in multi‐target single‐isocenter SRS applications are even more stringent, compared to the ones mentioned above. In 2/5 cases, plans that could be considered clinically unacceptable (clinical dose constraint violation in OARs) were obtained even for systematic leaf positional inaccuracies of as low as 0.19 mm (projected at the isocenter plane, corresponding to 0.06 mm at the MLC plane). In other words, this means that if the actual field size and beam segments at the isocenter plane deviate from the nominal ones by ≥0.38 mm (twice the leaf offset), clinically unacceptable VMAT‐SRS treatments might be administered. Although such tolerance levels might be stringent, a field size accuracy of 0.2 mm in MLC‐based SRS has been reported.^46^

In support of the results presented in the simulation study, a commercially available diode array and accompanying software were used in a plan QA check for a challenging multi‐target single‐isocenter VMAT‐SRS case. Varying the leaf offset parameter in the TPS and comparing with the measured dose distribution can reveal potential systematic leaf inaccuracies or sub‐optimal MLC modeling. More specifically, if a higher level of agreement between measurements and calculations is achieved after changing the clinically used leaf offset value, further investigation for systematic leaf positional errors or sub‐optimal MLC modeling is suggested. Nevertheless, this is a simple check that should be regarded as a practical tool to verify the MLC performance or ring a bell to further investigate systematic MLC positional errors. In a recent paper,^47^ systematic leaf errors of 0.5, 1, and 1.5 mm were intentionally introduced to both leaf banks in a stereotactic ablative body radiotherapy plan (often inducing a clinically significant dosimetric impact) and the authors investigated whether the clinical patient‐specific QA procedure is sensitive enough to detect these errors, within the context of a remote dosimetry audit test.^47^

A number of limitations of the present study are noteworthy. The study of the dosimetric impact of systematic MLC positional errors relied entirely on one MLC modeling parameter, that is, the leaf offset. If other parameters such as the leaf tip leakage, leaf transmission, and groove width, were also quantified, tolerance of uncertainties would potentially be different. Moreover, systematic offsets applied to all leaves and both leaf banks were only studied. Investigating random errors for individual leaves can be performed using the linac's log files.^48^, ^49^ However, log file‐based QA protocols can only detect leaf offsets compared to the expected positions and speeds. Systematic offsets to nominal (prescribed) positions are not accounted for. Nevertheless, results of the present study on systematic leaf errors, combined with published works^48^, ^49^ reporting on random errors can provide a comprehensive analysis on leaf positional uncertainties. Moreover, methodologies to determine installation‐specific uncertainty contributors are presented. Another limitation of this study is that presented results depended on the given spatial distribution, size, and shape of targets included in the analysis. Investigation of larger (>4 cc), smaller (<0.4 cc), or non‐spherical target volumes was not performed, although the induced dosimetric effect could also vary accordingly. Furthermore, cases with targets tangent to critical organs (e.g., brainstem metastasis^50^) were not considered, although not rare in clinical practice. It is expected that in such cases even more stringent tolerances may apply. Nevertheless, the design of this study focused on challenging single‐isocenter multi‐target VMAT‐SRS treatments but extreme cases were avoided in order not to jeopardize the generalization and applicability of the obtained results.

A stringent QA program is essential in order to minimize potential leaf positional uncertainties induced by systematic MLC leaf offsets. The main conclusion of this study is that acceptance and tolerance levels need to be tailored to VMAT‐SRS requirements, especially if small lesions are involved and/or are lying in close proximity to OARs. It was shown that acceptable uncertainties in conventionally fractionated treatments may potentially lead to clinically unacceptable single‐isocenter multi‐target VMAT‐SRS plans. In 2/5 cases, dose distributions that violated clinically meaningful dose constraints were obtained, if systematic leaf offsets of 0.19 mm (projected at the isocenter) are introduced to each leaf bank. Based on the results of the present study, a tolerance level of <0.38 mm in field size should be considered, which lies within the reported accuracy of a linac, commissioned for MLC‐based SRS delivery.^46^ A periodic verification or re‐adjustment of the MLC model parameters, such as the leaf offset, is crucial in order to ensure treatment delivery efficiency in challenging SRS applications. A simple QA check to verify the clinically used leaf offset MLC modeling parameter or reveal potential systematic errors was presented.

## 5 CONCLUSIONS

This simulation study focused on systematic leaf positional uncertainties, stemming from systematic mechanical inaccuracies of MLC leaf banks or sub‐optimal MLC modeling in the TPS. Emphasis was given in challenging multi‐target intracranial VMAT‐SRS treatments, utilizing a single‐isocenter.

Considering target and OAR dosimetry, systematic leaf offsets of 0.09 mm projected at the isocenter plane (0.03 mm at the MLC plane) are well‐tolerated, even for challenging cases where OARs lie in close proximity to targets. Plans that could be considered clinically unacceptable (clinical dose constraints violation) were obtained for projected leaf offsets of as small as 0.19 mm (0.06 mm at the MLC plane), corresponding to a discrepancy of 0.38 mm in field size, if static conventional fields had been used. The impact on target dosimetry is strongly associated with lesion volume.

A simple experimental QA check, based on diode array dosimetry, was performed to measure the delivered dose distribution for a challenging VMAT‐SRS case. Varying the clinically used leaf offset parameter and checking if a higher level of agreement between the measured and TPS‐calculated dose distributions can be achieved is an indication to further investigate potential systematic leaf errors or sub‐optimal MLC modeling in the TPS.

Acceptable and tolerance levels in systematic MLC uncertainties need to be tailored to VMAT‐SRS spatial and dosimetric accuracy requirements. A periodic verification or re‐adjustment of the MLC model parameters, such as the leaf offset, is crucial in order to ensure treatment delivery efficiency in challenging SRS applications.

## CONFLICT OF INTEREST

The authors declare that there is no conflict of interest that could be perceived as prejudicing the impartiality of the research reported.

## AUTHOR CONTRIBUTIONS

Study conception and design: Pantelis Karaiskos. Patient selection: Pantelis Karaiskos and Efi Koutsouveli. Contouring and treatment planning: Georgia Prentou, Eleni Prentou, and Efi Koutsouveli. Development of processing routines: Georgia Prentou and Evaggelos Pantelis. Data analysis: Georgia Prentou. Dose measurements: Nikolaos Yakoumakis and Chryssa Paraskevopoulou. Data interpretation: Pantelis Karaiskos, Evaggelos Pantelis, and Panagiotis Papagiannis. Preparation of figures and tables: Georgia Prentou and Eleftherios P Pappas. Literature review: Georgia Prentou, Eleftherios P Pappas, and Pantelis Karaiskos. Original manuscript preparation: Georgia Prentou, Eleftherios P Pappas, and Pantelis Karaiskos. Manuscript review and editing: all authors.
